# Low-Molecular-Weight Synthetic Antioxidants: Classification, Pharmacological Profile, Effectiveness and Trends

**DOI:** 10.3390/antiox11040638

**Published:** 2022-03-26

**Authors:** Mihaela Stoia, Simona Oancea

**Affiliations:** 1 Faculty of Medicine, “Lucian Blaga” University of Sibiu, 2A Lucian Blaga Street, 550172 Sibiu, Romania; medmuncii@dspsibiu.ro; 2 Department of Agricultural Sciences and Food Engineering, “Lucian Blaga” University of Sibiu, 7–9 Dr. Ion Ratiu Street, 550024 Sibiu, Romania

**Keywords:** synthetic antioxidants, natural-identical, antioxidant classification, pharmacological properties, health, disease, oxidative damage

## Abstract

Mounting research has been performed and published on natural antioxidants, more so than on synthetic ones, as key molecules that control oxidative damage and its pathway to disease. Since the discovery of vitamins, various fully synthetic or natural-identical compounds have been developed as stable small molecules translated into constantly active and completely controlled products which are widely exploited in the food and pharmaceutical industries. There is currently a debate within the literature about their mechanism of action, bioavailability, safety and real benefit for human health. Using a semiquantitative method and eligible criteria of selection, this review aimed to provide a very useful classification of antioxidants and a comprehensive cross-disciplinary description of 32 approved synthetic/natural-identical antioxidants, in terms of regulatory, antioxidant mechanism of action, safety issues, pharmacological properties, effectiveness in human health, timeline and future trends. Enriched interpretation of the data was obtained from summary bibliometrics, useful to portray the “good antioxidant” within the period 1966–2021 and, hopefully, to encourage further research.

## 1. Introduction

The word “antioxidant” has become more and more popular in modern society, being widely promoted by mass media, due to the fact that consuming antioxidant compounds through diet provides health benefits. 

A simple Google search by typing the keyword “antioxidants” carried out on January 2022 produced 1.56 billion results. However, as noticed by Barry Halliwell, “the term antioxidant is widely used but rarely defined” [1]. A large number of definitions have been given to illustrate what an antioxidant is. Thus, according to the *Dictionary of Pharmaceutical Medicine*, antioxidants are “substances (e.g., vitamin C, sulphites, ascorbyl palmitate, alkyl gallate, hydrochinone, tocopherols) used in pharmaceutical formulations to inhibit the reaction with oxygen in the surrounding atmosphere; they can react with free radicals to form stable or meta-stable products, thus terminating the oxidation reaction (radical scavenger) in contrast to pro-oxidant agents that increase oxidative stress” [2]. According to the *Oxford Dictionary of Science*, antioxidants are “substances that slow the rate of oxidation reactions. Various antioxidants are used to preserve foodstuffs and to prevent the deterioration of rubber, synthetic plastics, and many other materials. Some antioxidants act as chelating agents to sequester the metal ions that catalyze oxidation reactions. Others inhibit the oxidation reaction by removing oxygen free radicals” [3]. According to the *Handbook of Food Preservation*, “in a broad sense, antioxidants are all substances that can protect materials (not only foods) against autoxidation, irrespective of the mechanism of action. More exactly, such compounds should be called oxidation inhibitors, and only those substances that inhibit oxidation by reaction with free radicals should be called antioxidants” [4].

By far, a much larger number of works were performed and published on natural antioxidants than on synthetic ones. A search on the topic “natural antioxidants” or “synthetic antioxidants” from one of the world’s most trusted global citation databases, Web of Science, for the period 2000–2022 (accessed by 21 January 2022) showed 42,577 results on the topic “natural antioxidants” consisting of articles, proceedings papers, books and chapters, and meeting abstracts published in English, of which ~33% were published in Food Science Technology/Nutrition/Dietetics, and ~21% in Pharmacology/Pharmacy/Chemistry Medicinal, mostly published by researchers from China, India and the USA. Regarding the topic “synthetic antioxidants”, it produced only 9692 results of same type of papers published in English (~77% less than for natural antioxidants), of which ~28% were published in Food Science Technology/Nutrition/Dietetics, and ~19% in Pharmacology/Pharmacy/Chemistry Medicinal, mostly published by researchers from India, the USA and China. Despite this, synthetic antioxidants have been successfully used for their high efficacy, low cost and stability in products such as foods, pharmaceuticals or cosmetics; the interest in them has declined because of some safety and health concerns [5].

Vitamins played an essential role in nutrition research and control of the major deficiency syndromes in the 20th century, while the present interest relies on the debate of whether mortality is influenced in the long-term administration of antioxidant vitamins. In this respect, the critical review of Bjelakovic et al. concluded that consuming supplements based on antioxidant vitamin A, C and selenium did not influence mortality neither in healthy individuals, nor in patients; on the contrary, vitamin E was found to increase mortality [6]. Later, some researchers described the effectiveness of the mineral compound zinc sulfate in treating thalassemia and sickle cell diseases, as well as in preventing red blood cell dehydration, by decreasing the number of clinical infections and the number of crises, respectively [7,8].

The process of market authorization of medicines is a laborious one compared to that required for dietary supplements, being the subject of continuous evaluation in terms of safety, efficacy and extended usage. This is the reason why some products have been withdrawn, while others have been found useful in a more extended therapeutic area. Considering the antioxidant compounds, it is questionable if administration depends on evaluating the “oxidative stress status” of individuals or not, because in the absence of an index or a measurable biomarker, the “antioxidant dose” remains unclear [9]. 

Because new knowledge in the field of free radical biology is a great opportunity for chemists to investigate compounds that might block or activate the function of selected target molecules, there is a high requirement for further development of synthetic antioxidants. However, the current approved antioxidants are not completely portrayed and clearly classified in previous research, and there are disagreements regarding their evidence-based effectiveness in humans. Therefore, in this review, we focus on the chemical and pharmaceutical profiles of 32 principal low-molecular-weight synthetic antioxidants based on the long-held antioxidant hypothesis. Enriched interpretation was given to systematic data by using a semiquantitative method to highlight the scientific interest in the context of strengths and weaknesses of the evidence-based medicine when evaluating the effectiveness of these compounds on human health and estimating future trajectories. Moreover, the impact on public health in terms of epidemiological associations with overall-cause mortality was also discussed. The aim of our multiobjective approach, bridging chemistry with pharmacology, clinical medicine and public health, is to provide insight on synthetic antioxidants as a useful tool for multidisciplinary specialists and possibly health policy actions.

## 2. Study Design

A semistructured method was used to conduct this study by combining the narrative review [10] with summary bibliometrics—in particular a co-word analysis [11]. 

The study started with the identification of approved and experimental synthetic antioxidants using the online databases DrugBank and PubChem, followed by the keyword search using several multidisciplinary databases (SCOPUS, Web of Science), publisher databases (ScienceDirect, SpringerLink) and medicine libraries (PubMed, Cochrane Library) for relevant original articles, review-type articles, books and scientific reports. Search terms such as “antioxidant”, “synthetic antioxidant”, “approved antioxidants”, “antioxidant drugs” and “nano-antioxidants” were mainly used. The results were synthesized as much as possible in tables, charts and graphic design. Systematic data on approved synthetic low-molecular-weight antioxidants, in terms of identification, principal characteristics and biological effects, were translated into a list. Moreover, a comprehensive classification of antioxidant molecules was proposed on the basis of eight eligible criteria: mode of action, mechanism, molecular size, solubility, intended use, occurrence, biological defense and location. Regarding the summary bibliometric analysis, the quality of evidence regarding synthetic antioxidants relevant to health was the main selection criteria applied for the data mining process from systematic reviews and randomized clinical trials, respectively. Analysis was performed on articles published in the 55-year period 1966–2021 and expressed in productivity indicator (number of publications). 

**Figure 1:**
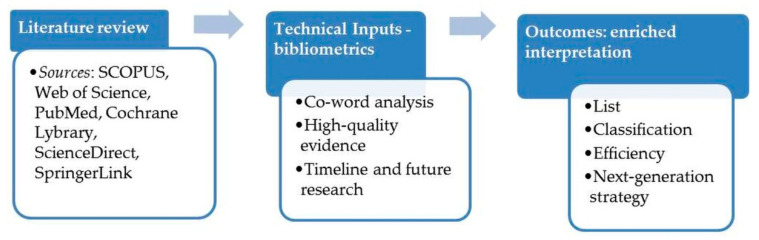
Overview of the Synthetic Antioxidants Progression Model.

The workflow is summarized in Figure 1. 

We expect that the two-stage “antioxidant description−antioxidant effects on human health” review matrix will provide a reasonably comprehensive and factual synthesis of knowledge within the context of strengths and limitations of and insight into low-molecular-weight synthetic antioxidants.

## 3. Approaches to Reduce Oxidative Damage

In biological systems, oxidative stress is produced when reactive oxygen species (ROS) or reactive nitrogen species (RNS) are generated in larger quantities than can be eliminated or stabilized by endogenous antioxidants, a process connected to cellular toxicity and pathologies such as cardiovascular, inflammatory, cancer and neurodegenerative [9].

The ROS, superoxide (O_2_^•−^), hydrogen peroxide (H_2_O_2_), hydroxyl radical (^•^OH), singlet oxygen (^1^O_2_), RNS, nitric oxide (^•^NO) and peroxynitrite (ONOO−), generated due to the enzymatic action of xanthine oxidase, monoamine oxidase, NADPH oxidase, nitric oxide synthase, electron transport chains, hemoglobin, auto-oxidation or photochemical reactions, may produce other unstable species called free radicals (e.g., lipid, protein or DNA radicals) [12,13]. 

The main approach to designing new antioxidants is based on the control of two reactions of radical species [12] by different mechanisms, as follows:

(1) Control of the chain initiation oxidation reactions: (i) preventing O_2_^•−^ formation through inhibition of xanthine oxidase (allopurinol); (ii) scavenging O_2_^•−^ (ascorbic acid) or ^•^OH; (iii) chelating metal ions such as Fe^2+^ (ascorbic acid)—**preventive antioxidants**;
(2) Control of the chain propagation oxidation reactions: terminating/breaking the auto-oxidative chain reactions (probucol, α-tocopherol and its derivatives)—**proper antioxidants/chain-breaking antioxidants**.

Other approaches to designing new synthetic antioxidants are based on the fact that radical species mediate signal transduction and gene expression. Therefore, some antioxidants (N-acetyl cysteine) influence gene expression by preventing the activation of a transcription factor, NF-*k*B, which is triggered by H_2_O_2_. Other strategies include searching activators of the activity and expression of endogenous antioxidant enzymes (superoxide dismutase, catalase, glutathione peroxidase) or inhibitors of the activity of pro-oxidative enzymes (NADH oxidase, xanthine oxidase, lipoxygenase). Lipoxygenases are enzymes containing non-hem iron which catalyze the oxidation of polyunsaturated fatty acids, being inhibited by different antioxidants or iron chelators, such as ascorbyl palmitate, α-tocopherol and phenolics. Furthermore, therapeutic antioxidant drugs, in particular lipophilic, may act by multiple mechanisms, e.g., by scavenging ROS plus inducing physical effects on cell membranes [12]. The expression of proteins with antioxidant, anti-inflammatory and other cytoprotective properties induced by the activation of transcription factor nuclear factor-erythroid 2 p45-related factor 2 (NRF2) is one important mechanism exploited in current clinical research on several small-molecule NRF2 activators, e.g., cyclic cyanoenones [14,15].

There are countless research works dealing with the understanding of the mechanism of action of antioxidants in chemical or biological systems which can help findings of new antioxidants, in particular natural ones, generally considered safer than synthetic antioxidants [16,17].

## 4. Classes of Antioxidants

There is mounting research dealing with different antioxidant molecules, which were classified in several ways, the majority of the studies considering the two most frequently used criteria, the antioxidant mechanism of action (primary and secondary antioxidants) and catalytic issues (enzymatic and non-enzymatic antioxidants) [5,18,19,20,21,22,23,24]. Furthermore, scientists, industry and media representatives, and society/consumers alike often use the words “natural” and “synthetic” when referring to antioxidants. Most people give a negative connotation to the word “synthetic” in relation to a general fear of chemicals in the absence of scientific knowledge. Natural antioxidants are produced by living organisms, such as microorganisms, fungi, plants and animals, generally for their own benefits [5]. Synthetic antioxidants are molecules of various chemical structures produced by experts in the industry, for the benefit of mankind [5]. Both natural and synthetic antioxidants exhibit a certain level of toxicity [5].

Herein, we provide a new classification of antioxidants, based on eight criteria: mode of action, antioxidant mechanism, molecular size, solubility, intended use, origin/occurrence, biological defense and location, as illustrated in Table 1. Furthermore, researchers’ studies concerning antioxidants may be guided by considering the target biomolecule they protect; a great number of studies aim to develop molecules that protect lipids (tocopherols, carotenoids, ascorbate, phenolics, glutathione peroxidase, lipoic acid), proteins (preventive antioxidants, tocopherols, phenolics) or DNA (SOD, glutathione peroxidase, reduced glutathione, cysteine, vitamins) [12].

Antioxidants work synergistically, protecting cells against oxidative damage in biological systems [25].

A considerable number of antioxidants are low-molecular-weight molecules, either natural or synthetic, and either hydrophilic or lipophilic, which efficiently scavenge oxygen, nitrogen- and carbon-centered radicals or inhibit chain oxidation reactions; at higher concentrations, such molecules may become pro-oxidants [12].

Generally, synthetic antioxidants are more active and pure than natural ones and possess constant antioxidant activity; on the other hand, they must pass criteria of nontoxicity and safety required by regulatory agencies prior to their marketing [5]. Natural-identical antioxidants combine the advantages of the fully synthetic (cheap, highly active, stable, reproducible properties) and the natural antioxidants (healthy). Some antioxidants provide both biological and antioxidant properties, being called *bio-antioxidants*, while others show only antioxidant properties without biological activity [26]. In some cases, the biological activity of an antioxidant may not be related to the antioxidant properties, as confirmed in disease models, the main challenge being that these effects (inhibition of cytokine and IL-1β production, antiproliferation) could contribute to an enhanced therapeutic drug [12].

## 5. Synthetic Low-Molecular-Weight Antioxidants: Properties and Pharmacological Effects

Synthetic small molecules with antioxidant activity may be used either as therapeutic agents, playing key roles as cellular antioxidants, or antioxidant additives. Such additives should be rationally used in pharmaceuticals, not to cover poorly formulated products but play key roles in the retardation of oxidation of active substances and excipients. Antioxidant additives are also used in the food industry with the purpose of retarding the oxidation of nutrients, in particular lipids and proteins. Lately, antioxidants have received attention as essential adjuvants in different types of disease.

Table 2 provides complete and useful data on synthetic and natural-identical antioxidants approved to be used in therapy, pharmaceuticals or foods, including regulatory items (FDA, Pharmacopeia standards) and safety issues related to potential adverse effects. Preservatives were not included in this study; for details, readers are invited to look up to the study of Franco et al. [27].

**Figure 2:**
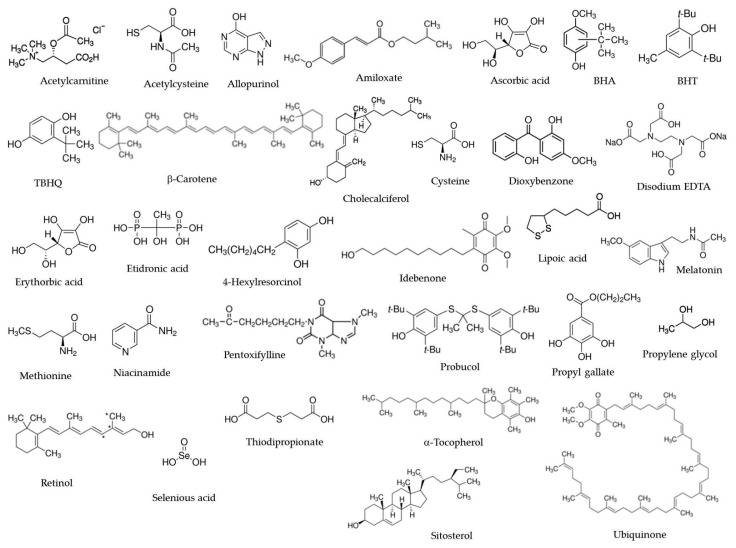
Chemical structure of approved principal synthetic low-molecular-weight antioxidants (alphabetical order).

Synthetic low-molecular-weight antioxidants are compounds of different chemical structures, such as alcohols/diols, phenols, benzene derivatives, isoprenoids, aldehydes, amino acid derivatives, indole-amines, fatty acid derivatives, etc., as depicted in Figure 2.

There are synthetic drugs designed for treating specific pathological disorders, e.g., dyslipidemias, which demonstrate in vivo or in vitro antioxidant activity, such as probucol. As shown in Table 2, there are several synthetic and natural-identical antioxidants (acetylcarnitine, acetylcysteine, ascorbic acid, β-carotene, cholecalciferol, α-lipoic acid, pentoxifylline, ubiquinone, zinc compounds) that have been considered a supportive and therapeutic option in patients with COVID-19, while other antioxidants such as β-sitosterol were investigated for SARS-CoV-2 infection (for references, see Table 2).

The process of discovery of new synthetic antioxidants as therapeutic drugs is a difficult one, as it requires the identification of bioactive chemical structure, the elucidation of the mechanism of action using different model systems, in vitro and in vivo, and the toxicological evaluation. The identification of new efficient antioxidant additives intended for pharmaceutical use requires complete physical-chemical characterization, solubility and stability studies and analytical strategies for individual pharmaceutical formulation. Next, the process of pharmacokinetics and pharmacodynamics of each drug candidate continues with experimental research (preclinical studies) on cell cultures (in vitro studies) and on lab animals (in vivo tests prior to human testing) to ensure safety for further clinical studies on human subjects (randomized clinical trials); the most accurate evidence has been provided by systematic reviews and meta-analysis of clinical trials. The marketed product will be further evaluated in terms of efficacy and adverse effects, with the updates and decisions being publicly available.

## 6. Effectiveness of Antioxidant Intervention in Human Health

**Figure 3:**
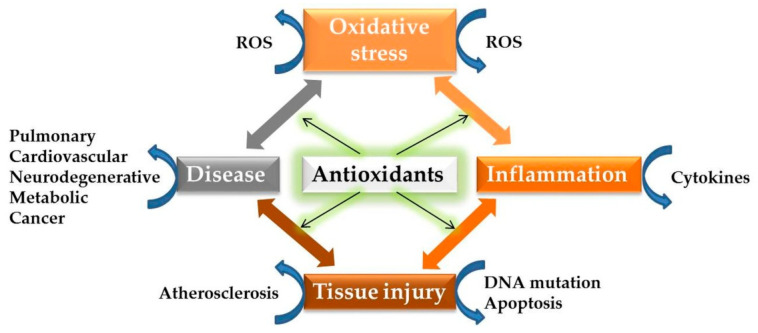
Effectiveness of antioxidants in the disease-specific pathways.

Within the defense system against oxidative stress, most exogenous antioxidants act mainly as radical scavengers that suppress chain initiation, or break chain propagation reactions, so-called primary antioxidants [22]. According to Forman et al., the assumption of the scavenging mechanism cannot be substantiated on a kinetic basis in vivo, thus explaining the limited effectiveness of small molecules in the therapeutic area [115]. An excessive increase in ROS may conduct to several pathological conditions, and finally to disease. This pathway consists of a positive feedback loop between oxidative stress and inflammation, with factors such as ROS and cytokines substantially contributing to inflammation-induced diseases, e.g., atherosclerosis, carcinogenesis, metabolic and neurodegenerative disorders, the process of which is illustrated in Figure 3. Understanding the spatial and temporal features of molecular signaling and cellular consequences within this cycle is helpful for the timing of intervention with targeted therapies [9].

Figure 3 points out the optimal benefit of antioxidant administration, which is included in the prevention protocol of certain diseases rather than in the therapeutic one. One reasonable argument could be the variable proportion of oxidative stress in the causality of diseases; thus, antioxidants are more often used to ameliorate symptoms.

Another reasonable argument in favor of antioxidant intervention in the first two stages of the disease-specific pathway could be the ability of some antioxidants to reduce inflammation, as confirmed for idebenone, explaining the pleiotropic protective effects via distinct signaling events [116]. In our opinion, the future belongs to a new understanding of the bioactivities of synthetic compounds at a molecular level, which could cut off both oxidative and inflammatory events.

Oxidative stress is not only involved in chronic inflammation [117], but also in the immune response to viral respiratory infections. Recent findings suggested elevated expression of genes involved in ROS production as responsible for the “cytokine storm”—a paradoxical hyper-inflammation in SARS-CoV-2 infection [118]. Early control of the “cytokine storm” is the key to reducing the severity of illness and mortality, as many authors agree [119]. Worldwide, efforts have been made to manage the COVID-19 pandemic, and paramount ongoing research is focused on multiple therapeutics, drug combination and antioxidants, among other targeted drugs. In this respect, N-acetyl cysteine alone or in combination with elastase inhibitors, melatonin, synthetic organoselenium compounds such as Ebselen or high doses of vitamins C or D_3_ are just a few examples of antioxidant pharmaceuticals proposed to be used in patients with severe COVID-19, and subject of registered clinical trials as well [120,121,122,123,124,125,126,127,128,129]. 

Time, route (oral/parenteral), and the form of administration (high/maintenance dose) are several key factors to be considered when evaluating the effectiveness and efficacy of these pharmaceuticals. In cancer, for example, concomitant administration of drugs with natural or synthetic antioxidants is not recommended because the effectiveness of chemotherapy is decreased [130]. In preterm infants, an increased risk of bleeding in the brain associated with extra vitamin E given I.V. has been demonstrated, while the risk decreased when the extra vitamin E was administrated by other routes [131]. Moreover, accurate determination of individual’s oxidative stress levels is recommended before prescribing the appropriate antioxidant supplement [132].

### 6.1. Dietary Antioxidants

Recent systematic reviews concluded that the overall quality of evidence considering antioxidant vitamins was low and few results were positive, as follows: (i) vitamin D_3_ may reduce the requirement for COVID-19 patients to be put on a ventilator [133]; (ii) vitamin C improved exercise-induced bronchoconstriction [134]; (iii) combination of vitamins C and E and β-carotene proved protection of the macula against age-related deterioration [135]; (iv) combination of vitamin C and β-carotene maintained the cognitive function in the elderly upon long-term use [136]. Furthermore, results from systematic meta-analyses on the relationship between vitamin supplementation and mortality showed no effect of vitamins C, D_3_ and E on all-cause mortality [137,138,139], while zinc supplementation in children under 5 years old significantly reduced the risk of all-cause mortality [140]. Cancer mortality (total) was significantly reduced by vitamin D_3_ supplementation, as demonstrated by an updated meta-analysis [141]. From the perspective of public health significance, mortality reduction proves the performance of proper prevention and/or treatment strategies as the major goal of increasing the quality of life. 

Research for new adjuvant therapies in rare diseases found the combination of vitamin E, vitamin C, zinc gluconate and selenomethionine to be effective in facioscapulohumeral muscular dystrophy [142]. This combination was approved as medicines under investigation in the European Union. The disease affects less than 1 in 10,000 people and is believed to be linked to oxidative stress.

### 6.2. Medicines in Use (Internal)

Since oxidative stress plays a crucial role in the pathogenesis of atherosclerosis and subsequently in the onset of cardiovascular disease, anti-atherogenic drugs have been developed to control oxidized low-density-lipoprotein cholesterol and implicitly endothelial damage and plaque formation. In this respect, several antioxidants such as probucol and the metal chelator EDTA were tested or reviewed, but results were inconsistent—no statistical significance was found in the case of probucol in the PROSPECTIVE clinical trial [143], while insufficient evidence was observed to determine the effectiveness or ineffectiveness of the chelation therapy [144]. 

### 6.3. Cosmeceuticals

Dietary antioxidants were found to be effective in cosmeceutical applications, in particular some combinations of vitamins C and E intended for skin anti-aging and skin radiance [145,146]. Topically applied melatonin provided effective protection against the harmful effects of UV radiation and skin damage [147], being also recommended for the treatment of androgenic alopecia in the new formulation with lipid nanocarriers [148]. Alongside other advances in this field, the new disciplines, skin chronopharmacology and preventive skin medicine, have been shaped.

### 6.4. Antioxidant Additives

Synthetic antioxidant additives have been used in food, pharmaceuticals or cosmetics in order to protect nutrients or bioactive compounds against oxidation and thus to extend the shelf life of products. The main approach to develop novel antioxidant additives seems to be the chemical manipulation of natural antioxidants, in particular in terms of lipophilicity (e.g., nonpolar esters of polyphenols/phenolic acids/caffeic acid, substituted by aminophenol analogues) [149]. The synthesis of innovative antioxidant additives is required because of the harmful effects exhibited by some of the most widely used antioxidants, as documented for BHA, BHT and TBHQ [31,44].

A complete review on food antioxidant additives, natural and synthetic, in relation to their properties, mechanism of action, legislation and applications have been published by Carocho et al. [150]. 

Excipients with antioxidant properties play a fundamental role in pharmaceutical formulations, as it preserves the efficacy, safety and stability of the bioactive compound. However, the added antioxidant itself should prove efficacy and lack of toxicity. The results of recent research suggest that the synthetic additive propylene glycol, used in the fourth generation of e-cigarettes, may induce airway epithelial injury and tissue hypoxia in young tobacco smokers, and also transiently impair arterial oxygen tension in heavy smokers during heating [151]. In our opinion, this issue could be a starting point for more policy action in regulating electronic cigarettes; thus, further evidence is required. 

Common synthetic and natural-identical antioxidant additives used in cosmetics are BHA, BHT, TBHQ, propyl gallate, ascorbic acid, β-carotene, retinyl palmitate, tocopherols, cysteine, niacinamide, acetylcysteine, dioxybenzone, EDTA and hexylresorcinol [32].

### 6.5. Summary Bibliometric Data and Future Trends

**Figure 4:**
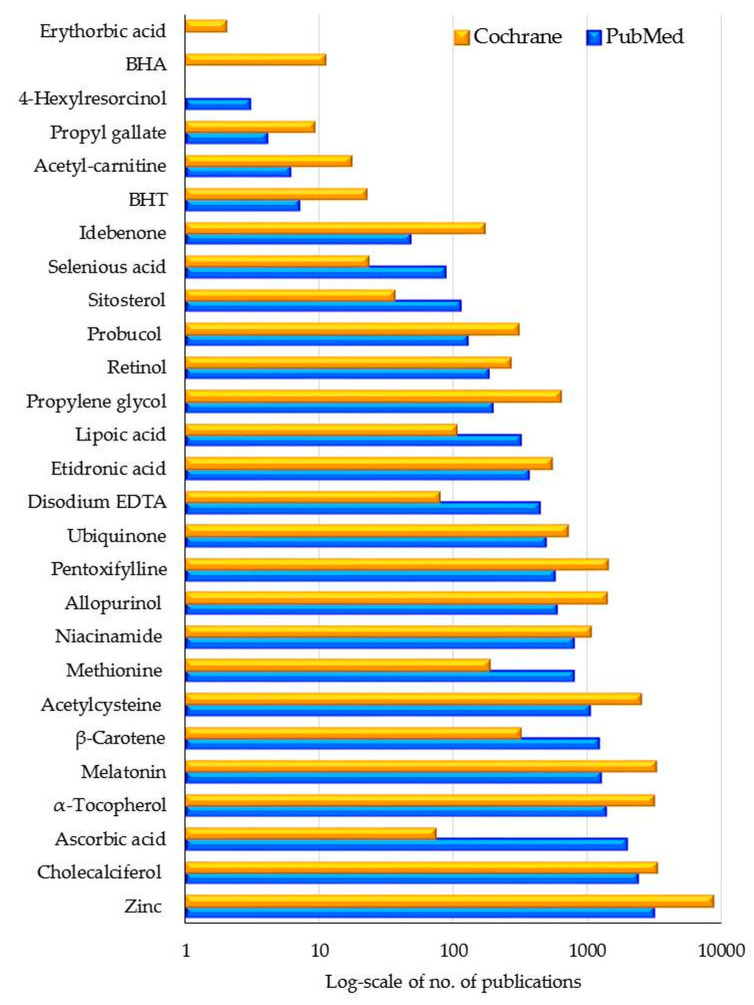
The number of publications on synthetic antioxidants as resulted from the bibliometric analysis of systematic reviews and randomized clinical trials using Cochrane Library and PubMed database (log-scale plot).

Bibliometric analysis of the global research output in the field of synthetic antioxidants selected by us and described in Table 2 consisted of 17,260 articles extracted from PubMed, 27,794 articles extracted from Cochrane Library, respectively. Dietary antioxidants were of high interest in human health, particularly zinc and cholecalciferol, as shown in Figure 4. Five of the total 32 antioxidants were not the subjects of medical research dealing with the synthetic approaches and were not included in Figure 4, namely amiloxate, TBHQ, cysteine, dioxybenzone, and thiodipropionate. According to the results by year provided by PubMed, this volume of literature evolved with time as follows: in the last two decades paramount research was dedicated to zinc, cholecalciferol, ascorbic acid, melatonin, acetylcysteine, methionine, niacinamide, allopurinol, ubiquinone, lipoic acid, propylene glycol and sitosterol; between 2000 and 2014, research was representative for α-tocopherol, retinol and etidronic acid; and β-carotene was of high interest until 2004.

Most of the research started in 1966–1975; ubiquinone and retinol raised interest in the 1980s; research on idebenone and selenious acid began in the 1990s, while propyl gallate has been associated with human benefits since 2005.

More than a technical input, this analysis is intended to add contextual value of a „good antioxidant” and their potential utility in health policy, industry, communities and the research field. Looking for scientific literature, it can be concluded that research on the vitamins continues to evolve from their discovery 100 years ago [152]. 

**Figure 5:**
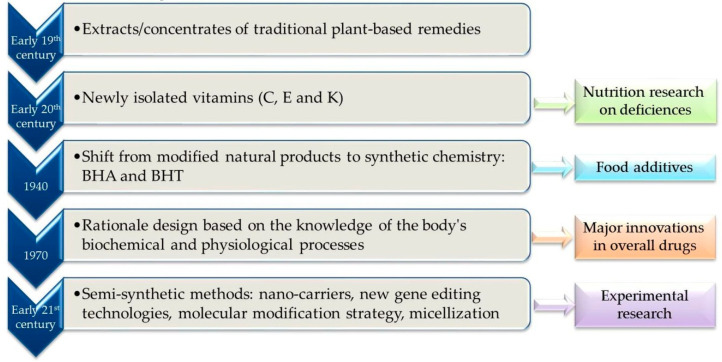
Timeline of important discoveries in the field and ongoing research instruments.

Given the historical interest of direct application of chemical research to medicine [153], our understanding of the rational design of molecular-targeted compounds and the next strategies may be enriched with a timeline of research in the field as illustrated in Figure 5.

The current research on synthetic antioxidants mainly follows three objectives, as follows: (1) enhancement of the bioavailability and efficacy of oral pharmaceuticals through novel formulations, e.g., nano-antioxidants; (2) modification or improvement of the chemical structures/moieties of existing compounds; and (3) design and synthesis of novel derivatives using a building block molecule, such as the well-known BHT. Despite the potential harmful effects of BHA, BHT and TBHQ due to their cytotoxic metabolites [44], experimental research is challenging because some authors found that BHA, TBHQ and 0.5/2 combination of BHT/BHA act as inhibitors of TNF and protect cells against apoptosis [154,155]. Indolinonic hydroxylamine emerged as a promising synthetic antioxidant [156,157], as shown by in vivo models of diabetic complications [158]. The list of novel compounds is open and may ground future research on effectiveness as well as debatable issues. Some of them could be medicines under evaluation, e.g., omaveloxolone, which is a synthetic oleanane triterpenoid to be used in patients with Friedreich’s ataxia [159], or bardoxolone-methyl for chronic kidney disease, type 2 diabetes mellitus and diabetic nephropathy—both acting as NRF2 activators [160]. The approved dimethyl fumarate and diroximel fumarate as medicines in the European Union have been developed as immunomodulators to treat relapsing forms of multiple sclerosis, but they also work via NRF2, fostering antioxidative pathways and increasing glutathione levels [161,162,163,164].

Several strategies have been developed since the 1980s aiming to find the best fit formulation of bioactive compounds, in terms of stability, solubility and efficacy. In the last decade, nanotechnology appears to be a very promising approach to develop novel antioxidant agents, as nanoparticles can be better controlled in terms of appropriate usage. Nano-antioxidants may refer to nanoparticles functionalized with antioxidants, the encapsulation of antioxidants into nanocarriers or nanoparticles/nanomaterials with intrinsic antioxidant properties such as metals and carbon structures-C_60_ and their derivatives, carbon nanotube and graphene [165,166,167,168]. For detailed information on nano-antioxidants, readers are invited to look up to the study of Khalil et al. [169]. The formulation of micellar nanoparticles for improving solubility should also be mentioned as another advance in the field, which has been applied to Coenzyme Q_10_ and silymarin [170,171]. To our knowledge, a single clinical trial was dedicated to nano-antioxidants, regarding the efficacy of an antioxidant nanogel to be used in oral surgery [172]. Nanoparticles are expected to play a beneficial role in medicine, but their safety should be carefully evaluated, and further research is required.

## 7. Conclusions

Mounting research has been conducted on natural antioxidants as important molecules to control oxidative damage, more so than on synthetic ones. However, a better classification was needed to enable informed interpretation and meaning of natural, natural-identical, fully synthetic and nano-antioxidants. A focus on principal synthetic antioxidants revealed that most (75%) were developed as pharmaceuticals and medicines with pleiotropic effects. Enriched interpretation of efficacy was given by summary bibliometrics, highlighting zinc and cholecalciferol as “good antioxidants” in terms of research productivity; on the basis of high-quality evidence, their effectiveness in human health was particularly by contributing to child and cancer mortality reduction. These findings are of major public health significance regarding the pharmacological success of dietary antioxidants in improving the quality of life. 

New research opportunities may come from idebenone and novel synthesized compounds, such as NRF2 activators possessing antioxidant, anti-inflammatory and cytoprotective properties expected to better control the oxidative damage at the molecular level.

With respect to antioxidant additives, the safety of BHA, BHT, TBHQ and PEG poses a potential public health concern; therefore, further investigations are required to substantiate community awareness and even policy action. 

We hope that this approach will contribute to a rational pharmaceutical perspective and expect to revive the research on synthetic or natural-identical antioxidants.

## Figures and Tables

**Table 1:** Complete classification of antioxidant molecules according to mechanistic, physical-chemical, biological and application criteria.

Mode of Action	Mechanism	Molecular Size	Solubility	Applications	Origin/Occurrence and Others			
*True antioxidants* ascorbic acid, tocopherols, BHA, BHT, PG	*Primary/Chain-breaking* catalase, glutathione peroxidase, SOD, tocopherols, BHA, BHT, TBHQ, PG	*Small* ascorbic acid, tocopherols, uric acid, ubiquinol, BHA, BHT, PG, etc.	*Hydrophilic* ascorbic acid, flavonoids, glutathione transferins, uric acid	*Health/Multipotent* dietary supplements (vitamins), therapeutic drugs intended for various diseases related to oxidative stress (idebenone, acetylcysteine, allopurinol, etc.)	*Natural*	*Endogenous*	**Biological defense**	*Enzymatic* catalase, glutathione peroxidase, SOD
								*Non-enzymatic* β-carotene, Co-enzyme Q10, glutathione, α-lipoic acid, tocopherols, uric acid
							**Location**	*Intracellular* catalase, glutathione peroxidase, SOD1/2
								*Extracellular* ascorbat, β-carotene, reduced glutathione, SOD3, uric acid
								*Membrane-associated* α-tocopherol, lutein, zeaxanthin
						*Exogenous* various chemical structures, e.g., vitamins (A, D_3_, E, B_3_, C), pro-vitamins (carotenoids), minerals (Se, Zn), polyphenols, organosulfur compounds (Cys, Met, glutathione)		
*Antioxidants synergists* improve the effects of true antioxidants, e.g., sodium edetate, preservatives (organic acids, lecithin)	*Secondary/Preventive* metal chelators, ^1^O_2_ quenchers, oxygen scavengers, primary antioxidants regenerators, vit C, EDTA, glutathione reductase, retinol, selenium, thiodipropionate	*Large* enzymes, proteins	*Hydrophobic/Lipophilic* ascorbyl palmitate, carotenoids, tocopherols, ubiquinol, BHA, BHT, PG, TBHQ	*Additives/Functional agents* maintain quality of foods, pharmaceuticals, cosmetics, etc. E306, E310, E311, E312, E319, E320, E321	*Natural-identical* pure substances identical to natural ones, but synthetized by industry; ascorbic acid, β-carotene, tocopherols			
					*Fully synthetic* EDTA, BHA, BHT, PG, TBHQ, acetylcysteine			
					*Nano-antioxidants* various antioxidants delivered by nanoparticles such as oxides (magnetite, zinc oxides, copper oxide), mesoporous silica, chitosan, alginate, poly-D,L-lactide, polybutylcyanoacrylate, polycaprolactone, poly (lactic-co-glycolic acid)			

**Table 2:** List of 32 principal approved synthetic low-molecular-weight antioxidants used as technological additives, dietary supplements or therapeutic agents (alphabetical order).

Common Name/Alternative Names	IUPAC Name (PubChem)	Applications/Regulatory	Description	Ref.
Acetylcarnitine	(*R*)-3-Acetoxy-4-(trimethylammonio)butyrate	Drug Dietary supplements Antioxidant in dietary supplements /ATC N06BX12 UNII NDW10MX58T	**Pharmacological properties**: reduce oxidative stress in patients with Sickle Cell disease, positive effects on neurological disorders (psychostimulant, nootropic), neuropathies, potential antiviral (supportive and therapeutic option in patients with COVID-19 -clinical trial NCT04623619)	[21,28]
			**Antioxidant mechanism**: decreases the generation of free radicals, prevents peroxidation of lipids, and oxidation of proteins through a tyrosine kinase A receptor-mediated mechanism; increases intracellular glutathione levels	[29,30]
			**Safety issues**: LD_50_ (oral, rat) >5000 mg/kg; nonirritant to skin; nonmutagenic	[31]
Acetylcysteine (NAC)	(*R*)-2-Acetamido-3-sulfanylpropanoic acid	Drug Antioxidant in cosmetics /Eur. Pharmacopoeia	**Pharmacological properties**: antioxidant, mucolytic therapeutic agent, reduces the effects of acetaminophen overdose, prevents the contrast nephropathy; immune-modulating properties useful for the treatment and prevention of COVID-19	[32,33,34,35]
			**Antioxidant mechanism**: alteration of intracellular redox reactions, deacetylation to cysteine, which participates in the synthesis of the antioxidant glutathione (stimulates glutathione synthetase), scavenging different types of ROS	[36,37]
			**Safety issues**: hypersensitivity reactions	[21]
Allopurinol (International Nonproprietary Name by WHO)	4-Hydroxypyrazolo[3,4-d]pyrimidine	Therapeutic drug /Eur. and US Pharmacopoeia	**Pharmacological properties**: antioxidant, antigout, anticancer (leukemia, lymphoma)	[12,21]
			**Antioxidant mechanism**: inhibitor of xanthine oxidase, prevents O_2_^∙^^−^	[12]
			**Safety issues**: rushes, lymphadenopathy, leucopenia or leukocytosis, eosinophilia, arthralgia, and vasculitis, hepatotoxic	[21]
Amiloxate (International Nonproprietary Name by WHO)	3-Methylbutyl (*E*)-3-(4-methoxyphenyl)prop-2-enoate	Cosmetics /US Pharmacopeia	**Pharmacological properties**: antioxidant, UV light absorber, anti-inflammatory, antimicrobial, antiedematous	[21]
			**Antioxidant mechanism of action**: free radical scavenger	[38]
			**Safety issues**: contact and photocontact allergen	[39]
Ascorbic acid (Vitamin C)	(2*R*)-2-[(1*S*)-1,2-Dihydroxyethyl]-3,4-dihydroxy-2*H*-furan-5-one	Dietary supplements Antioxidant in foods (*E300; sodium ascorbate E301; calcium ascorbate E302*), pharmaceuticals and cosmetics (ascorbyl palmitate) /FDA GRAS	**Pharmacological properties**: antioxidant, scavenger of free radicals, protection of DNA damage, involved in collagen synthesis, increases the intestinal absorption of iron, antiviral/immune-modulating properties useful for treatment and prevention of COVID-19, positive effects in age-related macular degeneration, neurological disorders, atherosclerosis, cancer	[21,40]
			**Antioxidant mechanism**: reducing agent, hydrogen donor forming a relatively stable ascorbyl-free radical Asc^−^^∙^ (efficient electron donor in biological redox reactions) and dehydroascorbic acid; efficiently recycles other antioxidants (e.g., α-tocopherol, glutathione); ascorbic acid regenerates itself from Asc^−^^∙^ with NADH or NADPH-reductases; in the presence of copper and iron becomes pro-oxidant	[41]
			**Safety issues**: LD_50_ (oral, rat) 11,900 mg/kg, (IV, mouse) 518 mg/kg; large doses may result in hyperoxaluria and the formation of renal calcium oxalate calculi	[21,31]
Butylated hydroxyanisole (BHA)	mixture of 2-*t*-Butyl-4-methoxyphenol and 3-*t*-Butyl-4-methoxyphenol	Antioxidant in foods (*E320*), food packages, animal feed, pharmaceuticals and cosmetics /FDA (0.02% max. of fat/oil), GRAS	**Properties**: high antioxidant activity; increases the levels of liver glutathione and glutathione-S-transferase; not preferred for pharmacological use, due to safety concerns. It acts synergistically with BHT, PG	[42]
			**Antioxidant mechanism**: prevents lipid peroxidation acting as hydrogen donor and interrupting the free radical autoxidative chain reactions (the resulting oxidized phenolic ion is stabilized by the inherent resonance of the benzene ring)	[43]
			**Safety issues**: LD_50_ (oral, mouse) 2000 mg/kg; may cause rashes, hyperactivity; confirmed carcinogen (IARC group 2B)	[31,44]
Butylated hydroxytoluene (BHT)	2,6-Di-*t*-butyl-4-methylphenol	Antioxidant in foods (*E321*), food packages, animal feed, pharmaceuticals and cosmetics /FDA (0.02% max. of fat/oil), GRAS	**Properties**: high antioxidant activity; antiviral (inactivates lipid-containing viruses); not preferred for pharmacological use, due to safety concerns. It acts synergistically with BHA	[42,45]
			**Antioxidant mechanism**: prevents lipid peroxidation acting as hydrogen donor and interrupting the free radical autoxidative chain reactions (the resulting oxidized phenolic ion is stabilized by the inherent resonance of the benzene ring)	[43]
			**Safety issues**: LD_50_ (oral, rat) 890 mg/kg, (IP, mouse) 138 mg/kg, (IV, mouse) 180 mg/kg; suspected carcinogen (IARC group 3); human skin irritant; eye irritant; may cause rashes, hyperactivity	[31,44]
*t*-Butyl hydroquinone (TBHQ)	2-*t*-Butylbenzene-1,4-diol	Antioxidant in foods (*E319*), pet foods, animal feed, pharmaceuticals and cosmetics /FDA (limitation 0.02% of oil, 0.003% in dry sausage, 0.01% in rendered animal fat, 0.02% in margarine, 0.01% on fat in poultry)	**Properties**: antioxidant, antibacterial; not preferred for pharmacological use, due to safety concerns	[46]
			**Antioxidant mechanism**: is a nuclear factor E2-related factor 2 (Nrf2) agonist, increases the levels of glutathione	[47]
			**Safety issues**: LD_50_ (oral, rat) 700 mg/kg, (IP, rat) 300 mg/kg; tumorigenic and mutagen in experimental animals	[31,44]
β-Carotene (Provitamin A)	1,3,3-Trimethyl-2-[(1*E*,3*E*,5*E*,7*E*,9*E*,11*E*,13*E*,15*E*,17*E*)-3,7,12,16-tetramethyl-18-(2,6,6-trimethylcyclohexen-1-yl)octadeca-1,3,5,7,9,11,13,15,17-nonaenyl]cyclohexene	Antioxidant in foods [*E160a(i)*], animal feed and cosmetics Dietary supplements /FDA GRAS	**Pharmacological properties**: antioxidant, precursor of vitamin A, prevents age-related macular degeneration and ischemic heart disease, protects against cancer, antiviral; potential treatment option for COVID-19 with vitamin A	[21,48,49]
			**Antioxidant mechanism**: quenching of singlet oxygen ^1^O_2_, prevents lipid peroxidation by scavenging peroxide radicals	[50]
			**Safety issues**: nontoxic on skin; massive doses may cause yellowing of the skin; increase in cancer incidence by administration of high doses; LD_50_ >5000 mg/kg	[21,31]
Cholecalciferol (Vitamin D_3_)	(3*S*,5*Z*,7*E*)-9,10-Secocholesta-5,7,10-trien-3-ol	Dietary supplements Drug Pharmaceuticals /FDA GRAS	**Pharmacological properties**: antioxidant (despite some studies showing controversy), therapeutic potential in diseases related to oxidative stress, anti-ricket, enhances absorption of calcium and phosphorus along the small intestine, anti-inflammatory, cardioprotective, potential in the treatment of COVID-19 (clinical trial phase 1, 2019, NCT04407286)	[51,52,53]
			**Antioxidant mechanism**: reduces lipid peroxidation, induces antioxidant enzymes (SOD), stimulates the enzyme sirtuin 1 involved in reduction in oxidative stress and inflammatory response	[51]
			**Safety issues**: LD_50_ (oral, rat) 42 mg/kg; experimental teratogen	[31]
Cysteine	(2*R*)-2-Amino-3-sulfanylpropanoic acid	Antioxidant in foods (E920), pharmaceuticals and cosmetics Dietary supplements /FDA GRAS, Eur. and US Pharmacopoeia	**Pharmacological properties**: antioxidant, prevention of corneal ulceration after chemical burn, skin-whitening	[54]
			**Antioxidant mechanism of action**: reducing agent, precursor of reduced glutathione (GSH), chain-breaking antioxidant mechanism; in foods (fruits), L-Cys inhibits the activity of polyphenol oxidase and reduces browning by combination with reactive electrophilic quinones	[55,56]
			**Safety issues**: LD_50_ (IP, mouse) 1250 mg/kg, (IV, mouse) 771 mg/kg	[31]
Dioxybenzone	(2-Hydroxy-4-methoxyphenyl)-(2-hydroxyphenyl)methanone	Dermatological drug (sunscreen in cosmetics) /US Pharmacopeia	**Pharmacological properties**: antioxidant, UV light absorber, skin cancer chemopreventive	[57]
			**Antioxidant mechanism**: scavenging free radicals and relieving of oxidative stress related to cancer from UV exposure	[57]
			**Safety issues**: LD_50_ (oral, rat) >10 g/kg; nontoxic in single oral doses; nonirritating to rabbit eye or skin;	[32]
Disodium EDTA (disodium edetate)	Disodium 2-[2-[bis(carboxymethyl)amino]ethyl-(carboxymethyl)amino]acetate	Foods (*calcium disodium EDTA E385; Disodium EDTA E386*) Drug (Calcium disodium EDTA) Pharmaceuticals Cosmetics /FDA, Eur. and US Pharmacopoeia	**Pharmacological properties**: antioxidant, metal chelating, for treatment of lead poisoning (Calcium disodium EDTA) or hypercalcemia (disodium EDTA), potential use in COVID-19	[31,58]
			**Antioxidant mechanism**: reduces metal-induced free radical production, protects against DNA damage and lipid oxidation	[59]
			**Safety issues**: LD_50_ (oral, rat) 2 g/kg, (IV, mouse) 56 mg/kg, (IP, mouse) 260 mg/kg; experimental teratogen, reproductive effects; mutagenic data	[32,60]
Erythorbic acid (isoascorbic acid, isovitamin C)	(2*R*)-2-[(1*R*)-1,2-Dihydroxyethyl]-3,4-dihydroxy-2*H*-furan-5-one	Antioxidant in foods (*E315; sodium erythorbate E316*) and pharmaceuticals /FDA GRAS for E315	**Pharmacological properties**: antioxidant, antimicrobial, enhance iron bioavailability, antitumor	[31,61,62,63]
			**Antioxidant mechanism**: sodium erythorbate is a reducing agent acting similar to ascorbic acid despite it lacking vitamin C activity, and inhibits nitrite reaction in meat curing	[64]
			**Safety issues**: Nontoxic; mutagenic; causes DNA damage; LD_50_ (oral, mouse) 8300 mg/kg and LD_50_ (oral, rats) 18 g/kg (erythorbic acid); LD_50_ (oral, rats) >5 g/kg (sodium erythorbate)	[31,62]
Etidronic acid	(1-Hydroxy-1-phosphonoethyl)phosphonic acid	Drug (etidronate disodium) Antioxidant in pharmaceuticals and cosmetics /FDA, Eur. and US Pharmacopeia	**Pharmacological properties**: antioxidant, chelating agent for heavy metal ions, reduces osteoclastic activity (treatment of Paget’s disease, osteoporosis), increase the bone mineral density, inhibition of protein tyrosine phosphatase	[65,66]
			**Antioxidant mechanism**: calcium, iron chelator inhibiting the chondrocyte lipid peroxidation, suppress radical formation	[67]
			**Safety issues**: LD_50_ (oral, mouse) 1800 mg/kg; impairment of bone mineralization, hypercalcemia, esophageal cancer	[21,32]
4-Hexylresorcinol	4-Hexylbenzene-1,3-diol	Flavoring agent in food (*E586*) Topical antiseptic Cosmetics /Eur. and US Pharmacopeia	**Pharmacological properties**: antioxidant, antiseptic, antihelmintic, local anesthetic, antiviral (against parainfluenza virus type 3)	[21,68]
			**Antioxidant mechanism**: inhibits tyrosinase, increases glutathione levels preventing DNA damage, scavenging of peroxyl radicals and oxygen superoxide, reduces lipid and protein peroxidation	[69]
			**Safety issues**: LD_50_ (oral, rat) 550 mg/kg; LDLo (IP, mouse) 50 mg/kg, (subcut., mouse) 750 mg/kg; may irritate eyes, skin, respiratory tract; experimental reproductive effects	[31]
Idebenone	2-(10-Hydroxydecyl)-5,6-dimethoxy-3-methylcyclohexa-2,5-diene-1,4-dione	Drug (nootropic, antioxidant therapy) /EMA	**Pharmacological properties**: antioxidant, stimulates ATP production, neuroprotective (dementia, Alzheimer’s disease), treatment of visual impairment in patients with Leber’s Hereditary Optic Neuropathy; adjuvant for secondary effects of viral infection	[21,70,71]
			**Antioxidant mechanism**: scavenger of free radicals (superoxide) being recycled by mitochondrial and cytosolic reductase), inhibits lipid peroxidation in mitochondrial membrane; electron donor to mitochondrial electron transport chain	[70]
			**Safety issues**: LD_50_ (oral, rat) 10 g/kg, LD_50_ (IP, rat) 757–886 mg/kg	[70]
Lipoic acid	5-[(3*R*)-1,2-Dithiolan-3-yl]pentanoic acid	Drug (antioxidant therapy of diabetic neuropathy) Nutraceutical /Eur. and US Pharmacopeia	**Pharmacological properties**: antioxidant, analgesic, treatment of diabetic neuropathy, detoxification of mercury in brain cells, treatment of multiple sclerosis (clinical trial 2021) and schizophrenia (clinical trial 2021), potential antiviral including COVID-19	[21,72,73,74]
			**Antioxidant mechanism**: iron, copper chelation, scavenging ROS, the reduced form (dihydrolipoic acid) regenerates endogenous antioxidants (vitamin C, vitamin E, glutathione) repairing oxidative damage	[75]
			**Safety issues**: cholestatic hepatitis; LD_50_ (oral, rats) >2000 mg/kg; no mutagenic (Ames assay), not genotoxic (mouse micronucleus assay)	[21,76]
Melatonin	N-[2-(5-Methoxy-1H-indol-3-yl)ethyl]acetamide	Drug /US Pharmacopeia	**Pharmacological properties**: antioxidant, increases GABA and serotonin, efficient in sleep and autistic disorders, anticancer, sunburn prevention, potential positive effect on COVID-19 (clinical trial 2021)	[21,77,78]
			**Antioxidant mechanism**: direct free radical scavenging, stimulation of antioxidant enzymes, lowering of free radical generation by increasing oxidative phosphorylation in mitochondria and reducing electron leakage, protects against DNA damage	[79,80]
			**Safety issues**: LD_50_ (oral, mouse) 1250 mg/kg	[81]
Methionine	(2*S*)-2-Amino-4-methylsulfanylbutanoic acid	Dietary supplements Food and pharmaceutical flavoring agent Feed additive /FDA, Eur. and US, Pharmacopeia, FEMA GRAS	**Pharmacological properties**: antioxidant, antihepatotoxic, alternative to acetylcysteine in the treatment of acetaminophen overdose, metal chelating, precursor of cysteine; anticancer (clinical trial phase 2, 2021)	[21,82]
			**Antioxidant mechanism**: reduces ROS levels due to Met-sulfoxide reductase, activation of endogenous antioxidant enzymes, stimulating glutathione synthesis, heavy metal chelator	[83]
			**Safety issues**: LD_50_ (oral, rat) 36 g/kg, (IP, rat) 4328 mg/kg; as precursor of homocysteine, high doses may result in susceptibility of cardiovascular disease, type-2 diabetes, brain alterations	[31,84,85]
Niacinamide (Nicotinamide)	Pyridine-3-carboxylic acid amide	Dietary supplements Food fortification Feed additive (nutritional) Pharmaceutical intermediate Cosmetics /FDA GRAS, Eur., US Pharmacopeia	**Pharmacological properties**: antioxidant, prevents pellagra, precursor of coenzymes (NAD) involved in electron transfer reactions in the respiratory chain, skin stimulant, anticancer, reduces LDL, improves HDL, early Alzheimer’s disease treatment (clinical trial phase 2, 2021)	[21,31,32,86]
			**Antioxidant mechanism**: scavenging ^•^OH, ^1^O_2_ and superoxide O_2_^•^^−^, inhibits the initiation step of lipid peroxidation, increases glutathione levels, protects against both lipid and protein oxidation in brain	[86]
			**Safety issues**: LD_50_ (oral, rat) 3500 mg/kg, (IP, mouse) 2050 mg/kg, (subcut., rat) 1680 mg/kg	[31]
Pentoxifylline (International Nonproprietary Name by WHO)	3,7-Dimethyl-1-(5-oxohexyl)purine-2,6-dione	Drug /FDA, Eur. and US Pharmacopeia	**Pharmacological properties**: antioxidant, anti-inflammatory, immunomodulatory, treatment of peripheral vascular disorders, inhibits the production of the cytokine TNFα, treatment of Diabetic Kidney Disease (clinical trial phase 4, 2021); antiviral potential adjuvant in treatment of COVID-19 (clinical trial)	[21,87,88]
			**Antioxidant mechanism**: scavenges free radicals (^∙^OH), decreases lipid peroxidation	[89]
			**Safety issues**: LD_50_ (oral, rat) 1385 mg/kg	[90]
Probucol (International Nonproprietary Name by WHO)	2,6-Di-*t*-butyl-4-[2-(3,5-ditert-butyl-4-hydroxyphenyl)sulfanylpropan-2-ylsulfanyl]phenol	Drug US Pharmacopeia	**Pharmacological properties**: antioxidant, anticholesterolemic	[21]
			**Antioxidant mechanism**: inhibits lipid peroxidation	[91]
			**Safety issues**: LD_50_ (oral, mouse, rat) >5000 mg/kg	[92]
Propyl gallate (PG)	Propyl 3,4,5-trihydroxybenzoate	Antioxidant in food (*E310*), food packages, food-contact coatings, feed, pharmaceuticals and cosmetics /FDA (0.005% migrating from food pkg., 0.02% max. of fat or oil, GRAS, Eur. Pharmacopeia	**Pharmacological properties**: antioxidant, hepatoprotector, limited antibacterial and antifungal activity	[21,31]
			**Antioxidant mechanism**: hydrogen donor interrupting the free radical autoxidative chain reactions	[43]
			**Safety issues**: LD_50_ (oral, rat) 3.8 g/kg, (IP, rat) 0.38 g/kg; skin irritant; questionable carcinogen; experimental tumorigenic, teratogen, reproductive effects	[21,31,44]
Propylene glycol (PEG)	Propane-1,2-diol	Food emulsifier (*E1520*) Pet foods Agent for pharmaceuticals and cosmetics (viscosity control) /FDA GRAS, US and Eur. Pharmacopeia	**Properties**: antiseptic, humectant, efficient solvent and extractant of active ingredients including antioxidants	[31]
			**Antioxidant mechanism**: propylene glycol mannate sulfate induces the antioxidant enzymes (superoxide dismutase, glutathione peroxidase, catalase) eliminating the oxygen free radicals (study on hyperlipidemic rats)	[93]
			**Safety issues**: LD_50_ (oral, rat) 21 g/kg; ocular and skin irritant; no reproductive toxicity; not mutagenic, not carcinogenic	[31,94]
Retinol (vitamin A)	(2*E*,4*E*,6*E*,8*E*)-3,7-Dimethyl-9-(2,6,6-trimethylcyclohexen-1-yl)nona-2,4,6,8-tetraen-1-ol	Dietary supplements Cosmetics /FDA GRAS, Eur. and US Pharmacopeia	**Pharmacological properties**: antioxidant, role in vision, epithelial differentiation, growth, bone development, immunity, anticancer, anti-inflammatory, potential immunomodulator in COVID-19	[95,96]
			**Antioxidant mechanism**: scavenges lipid peroxyl radicals (LOO^∙^) by forming a stable *trans*-retinol radical intermediate, reduces DNA damage (studies in cancer therapy), stimulates endogenous antioxidant enzymes	[97]
			**Safety issues**: daily intakes of Vitamin A >50,000 IU in adults and 20,000 IU in infants and young children may cause toxic manifestations; LD_50_ (oral, rat, 10 day) 7910 mg/kg, (oral, mouse) 6060 mg/kg; experimental reproductive effects; hepatomegaly, visual disturbances	[98,99]
Selenious acid	Selenous acid	Pharmaceuticals Supplements /US Pharmacopeia	**Pharmacological properties**: antioxidant, enzymatic cofactor (glutathione peroxidase), anticancer, stimulates hemoglobin synthesis in erythroleukemia cell lines, immunomodulatory, prevention of atherosclerosis	[21,100]
			**Antioxidant mechanism**: selenium is a Cu^+^ chelator, inhibits DNA damage from ^•^OH radical, maintains the enzymatic activity of glutathione peroxidase	[16]
			**Safety issues**: LD_50_ 7 mg Se/kg for sodium selenite, 138 mg Se/kg for selenium sulfides (as formulated for anti-dandruff shampoos), and 6700 mg Se/kg for elemental selenium	[101]
Sitosterol	(3*S*,8*S*,9*S*,10*R*,13*R*,14*S*,17*R*)-17-[(2*R*,5*R*)-5-Ethyl-6-methylheptan-2-yl]-10,13-dimethyl-2,3,4,7,8,9,11,12,14,15,16,17-dodecahydro-1*H*-cyclopenta[a]phenanthren-3-ol	Dietary supplements Stabilizer in pharmaceuticals, cosmetics /Canadian Provisional DSL	**Pharmacological properties**: antioxidant, hypolipidemic, treatment of diaper rash, anti-inflammatory, antiapoptotic, anticancer, potential role as immunostimulant and inhibitor of SARS-CoV-2 spike glycoprotein	[21,31,32,102]
			**Antioxidant mechanism**: reduces liver lipid peroxidation in induced cancer colon, maintains the level of antioxidant enzymes (catalase, superoxide dismutase, glutathione peroxidase, glutathione reductase, glutathione S-transferase, reduced glutathione)	[103]
			**Safety issues**: LD_50_ (oral, mouse) >25,000 mg/kg; skin and eye irritant; nontoxic	[31]
Thiodipropionate	3-(2-Carboxylatoethylsulfanyl)propanoate	Foods (0.02% of fat or oil content of food) Food packages Pharmaceuticals Cosmetics (0.1% and rarely exceed 0.2%) /FDA	Properties: antioxidant, skin lightening. Acts synergistically with phenols	[104]
			**Antioxidant mechanism**: chain-breaking, decomposes hydrogen peroxide	[105]
			**Safety issues**: Dilauryl thiodipropionate: LD_50_ (oral, rat) >10.3 g/kg; no known toxicity; eye irritant Distearyl thiodipropionate: LD_50_ (oral, rat) >2500 mg/kg, (IP, rat) >2 g/kg;	[31]
α-Tocopherol (vitamin E)	(2*R*)-2,5,7,8-Tetramethyl-2-[(4*R*,8*R*)-4,8,12-trimethyltridecyl]-3,4-dihydrochromen-6-ol	Dietary supplements Antioxidant in food (*E306; α-tocopherol E307;* *γ-tocopherol E308; δ-tocopherol E309*), food packages, animal feed, pharmaceuticals and cosmetics /FDA GRAS	**Pharmacological properties**: high antioxidant activity, anticancer, prevents atherosclerosis, cardiovascular diseases and age-related macular degeneration	[106,107]
			**Antioxidant mechanism**: directly reacts and neutralizes ^•^OH, alkoxyl and lipid peroxyl (ROO^∙^) radicals stopping the ROS-induced damage; may be regenerated with vitamin C	[16]
			**Safety issues**: TDL_o_ (oral, rat) 7500 mg/kg	[31]
Ubiquinone (ubidecarenona, coenzyme Q 10)	2-[(2*E*,6*E*,10*E*,14*E*,18*E*,22*E*,26*E*,30*E*,34*E*)-3,7,11,15,19,23,27,31,35,39-decamethyltetraconta-2,6,10,14,18,22,26,30,34,38-decaenyl]-5,6-dimethoxy-3-methylcyclohexa-2,5-diene-1,4-dione	Dietary supplements Antioxidant in pharmaceuticals and cosmetics /US Pharmacopoeia	**Pharmacological properties**: antioxidant, cofactor in the mitochondrial electron transport chain, useful in the treatment of cardiovascular diseases, Parkinson’s, fibromyalgia, migraine, diabetes, adjuvant in COVID-19 (clinical trial phase 2, 2021, NCT04960215, 2020-005961-16)	[31,108,109]
			**Antioxidant mechanism**: reduces lipid peroxidation, increases antioxidant enzymes (catalase, superoxide dismutase, glutathione peroxidase)	[110]
			**Safety issues**: lethal dose >2000 mg/kg (oral, rat)	[111]
Zinc	Zinc	Dietary supplements Antioxidant in pharmaceuticals (zinc glycinate) Cosmetics /FDA FDA GRAS (zinc gluconate)	**Pharmacological properties**: antioxidant, enzyme activator, antimicrobial, antidiarrheic, anti-inflammatory, antitumor (clinical trial NCT04488783), antiviral including protection against COVID-19	[31,112,113]
			**Antioxidant mechanism**: induces antioxidant glutathione and enzymes (SOD, glutathione S-transferase, hemeoxygenase-1); protection of protein –SH groups; gene regulation (p53, NF-*k*B, AP-1)	[114]
			**Safety issues**: Zinc acetate: LD_50_ (oral, rat) 2510 mg/kg Zinc carbonate: TDLo (oral, mouse) 2800 mg/kg; experimental teratogen Zinc chloride: LD_50_ (oral, rat) 350 mg/kg; irritant, questionable carcinogen; experimental tumorigenic, teratogen, reproductive effects Zinc citrate: LD_50_ (oral, rat) >5000 mg/kg; experimental reproductive effects Zinc gluconate: LD_50_ (oral, mouse) 1290 mg/kg; experimental reproductive effects Zinc sulfate: LD_50_ (oral, rat) 2949 mg/kg; irritant, questionable carcinogen; experimental tumorigenic, teratogen, reproductive effects	[31]

## Abbreviations

ATC	Anatomical Therapeutic Chemical (ATC) classification system
BHA	Butylated hydroxyanisole
BHT	Butylated hydroxytoluene
COVID-19	Coronavirus disease 2019
DNA	Deoxyribonucleic Acid
DSL	Domestic Substance List
EDTA	Ethylenediamine tetra acetate
EMA	European Medicines Agency
FEMA	Flavor and Extract Manufacturers Association
FDA	The US Food and Drug Administration
GRAS	Generally Recognized as Safe
HDL	High-density lipoprotein
IL-1β	member of the Interleukin-1 family
IARC	International Agency for Cancer Research
I.P.	Intraperitoneal injection
IUPAC	International Union of Pure and Applied Chemistry
I.V.	Intravenous medication administration
LD_50_	50% Lethal dose
LDL	Low-density lipoprotein
NAD	Nicotinamide adenine dinucleotide, oxidized form
NADH	Nicotinamide adenine dinucleotide, reduced form
NRF2	Nuclear factor erythroid 2-Related Factor 2
PEG	Propylene glycol
PG	Propyl gallate
RNS	Reactive nitrogen species
ROS	Reactive oxygen species
SARS-CoV-2	Severe acute respiratory syndrome coronavirus 2
SOD	Superoxide dismutase
TDL_o_	Toxic Dose Low (the lowest toxic dose)
THBQ	*t*-Butyl hydroquinone
TNF	Tumor necrosis factor
UNII	Unique ingredient identifiers, generated by the joint FDA/USP Substance Registration System
WHO	World Health Organization
